# Adopting, implementing and assimilating coproduced health and social care innovations involving structurally vulnerable populations: findings from a longitudinal, multiple case study design in Canada, Scotland and Sweden

**DOI:** 10.1186/s12961-024-01130-w

**Published:** 2024-04-02

**Authors:** Gillian Mulvale, Jenn Green, Glenn Robert, Michael Larkin, Nicoline Vackerberg, Sofia Kjellström, Puspita Hossain, Sandra Moll, Esther Lim, Shioma-Lei Craythorne

**Affiliations:** 1 DeGroote School of Business, McMaster University, 4350 South Service Road, Suite 421, Burlington, ON L7L 5R8 Canada; 2 Florence Nightingale Faculty of Nursing, Midwifery and Palliative Care, King’s College London, London, United Kingdom; 3 Institute of Health and Neurodevelopment, Aston University, Birmingham, United Kingdom; 4 Region Jönköping County, Jönköping, Sweden; 5 The Jönköping Academy for Improvement of Health and Welfare, School of Health and Welfare, Jönköping University, Jönköping, Sweden; 6 Department of Health Research Methods, Evidence, and Impact (HEI), Faculty of Health Sciences, McMaster University, Hamilton, ON Canada; 7 School of Rehabilitation Science, McMaster University, Hamilton, ON Canada; 8 School of Health and Welfare, Jönköping University, Jönköping, Sweden; 9 SingHealth Office of Regional Health, Singapore Health Services, Singapore, Singapore

**Keywords:** Coproduction, Case study, Structurally vulnerable populations, Adoption, Implementation, Assimilation, Transformation

## Abstract

**Background:**

Innovations in coproduction are shaping public service reform in diverse contexts around the world. Although many innovations are local, others have expanded and evolved over time. We know very little, however, about the process of implementation and evolution of coproduction. The purpose of this study was to explore the adoption, implementation and assimilation of three approaches to the coproduction of public services with structurally vulnerable groups.

**Methods:**

We conducted a 4 year longitudinal multiple case study (2019–2023) of three coproduced public service innovations involving vulnerable populations: ESTHER in Jönköping Region, Sweden involving people with multiple complex needs (Case 1); Making Recovery Real in Dundee, Scotland with people who have serious mental illness (Case 2); and Learning Centres in Manitoba, Canada (Case 3), also involving people with serious mental illness. Data sources included 14 interviews with strategic decision-makers and a document analysis to understand the history and contextual factors relating to each case. Three frameworks informed the case study protocol, semi-structured interview guides, data extraction, deductive coding and analysis: the Consolidated Framework for Implementation Research, the Diffusion of Innovation model and Lozeau’s Compatibility Gaps to understand assimilation.

**Results:**

The adoption of coproduction involving structurally vulnerable populations was a notable evolution of existing improvement efforts in Cases 1 and 3, while impetus by an external change agency, existing collaborative efforts among community organizations, and the opportunity to inform a new municipal mental health policy sparked adoption in Case 2. In all cases, coproduced innovation centred around a central philosophy that valued lived experience on an equal basis with professional knowledge in coproduction processes. This philosophical orientation offered flexibility and adaptability to local contexts, thereby facilitating implementation when compared with more defined programming. According to the informants, efforts to avoid co-optation risks were successful, resulting in the assimilation of new mindsets and coproduction processes, with examples of how this had led to transformative change.

**Conclusions:**

In exploring innovations in coproduction with structurally vulnerable groups, our findings suggest several additional considerations when applying existing theoretical frameworks. These include the philosophical nature of the innovation, the need to study the evolution of the innovation itself as it emerges over time, greater attention to partnered processes as disruptors to existing power structures and an emphasis on driving transformational change in organizational cultures.

**Supplementary Information:**

The online version contains supplementary material available at 10.1186/s12961-024-01130-w.

## Background

Growing recognition by governments internationally of the need to involve the perspectives of people using public services when designing, delivering and improving those services has been described as a Participatory Zeitgeist reflecting the “spirit of our time” [1, 2(p247)]. Researchers and designers have developed various approaches drawn from different disciplines and using different labels (for example, codesign, cocreation, coproduction) that align with principles in the citizen engagement literature [3]. These approaches recognize that service users have experiences and assets and can contribute to service design and delivery along with professional expertise, rather than simply being passive recipients of services designed and delivered by others [3, 4]. While these approaches can be used with anyone, they have been increasingly applied to promote the inclusion of structurally vulnerable populations in the design and delivery of innovative health and social care services that seek to support them.

While coproduction has the potential to reform inequitable structures and social processes, excluding vulnerable groups or involving them in a tokenistic manner may unintentionally reinforce existing power imbalances [4, 5]. For example, gaps have been noted between the rhetoric of service user involvement in international mental health policy and the readiness to adopt such policies in practice [6]. Challenges have also been noted in incorporating the voices of individuals with complex needs in improving care coordination across health and social services [7].

Despite increasing attention to coproduction in the literature and practice, knowledge gaps exist with respect to the implementation of coproduction involving vulnerable populations in different contexts [8–10]. An international symposium of coproduction researchers and people with lived experience held in Birmingham, England in 2017 identified the need for research to understand how exemplary coproduction innovations involving structurally vulnerable groups originated and their assimilation into routine practice [11]. To our knowledge, established implementation science models have yet to be applied to coproduction, where service users and service providers are cocreating innovations during the process of implementation [12].

In this paper, we present findings from a longitudinal case study exploring the factors and processes that influence the adoption, implementation and assimilation of three diverse coproduced public service innovations involving structurally vulnerable groups. We explored the perspectives of strategic leaders involved in advancing coproduction processes involving vulnerable groups. Our analysis proceeds through the lens of existing frameworks from the literature to discuss the outer context (economic, social, political, geographical), inner context (organizational and community considerations), individual factors, innovation features and process considerations [13].

### Conceptual foundations: coproduction, structural vulnerability and implementation processes

Coproduction: Coproduction has been defined as “… involvement of public service users in the design, management, delivery and/or evaluation of public services” [4]. A core feature of coproduction approaches is that they are applied in a flexible manner, dynamically and innovatively responding to local needs and context [14].

Structural vulnerability: We adopt the term structurally vulnerable populations to recognize that vulnerability is not inherent in these populations but rather in the social, economic and political systems in which they are embedded [15, 16]. Examples include individuals who may require multiple health and/or other public services, including people with complex and intersecting health needs (for example, heart failure and dementia) along with poverty, homelessness and/or being members of newcomer or racialized groups. Structural barriers (for example, lack of trust, language, cultural, scheduling, financial) and power relations may prevent them from engaging in coproduction.

Adoption, implementation and assimilation: We draw on and combine elements from three theoretical frameworks to guide this research. The first is the Diffusion Of Innovation (DOI) model [17], which identifies how political, social, economic, cultural, and organizational factors and processes affect fidelity and adoption during the diffusion of service innovation. The second is the Consolidated Framework for Implementation Research (CFIR) [18, 19], which demonstrates the importance of contextual factors at multiple levels (external context, internal context, innovation features, processes and individual characteristics) in shaping the implementation of service improvements. The third is Lozeau et al.’s (2002) compatibility gaps [20], which characterize different forms of assimilation of innovations into routine practice [20, 21]. Based on these frameworks, we define innovation as a novel set of behaviours, routines and ways of working that are directed at improving health outcomes, administrative efficiency, cost effectiveness or users’ experiences, and that are implemented by planned and coordinated action [20]. We define adoption as the incremental considerations and progressive individual and collective decision-making from pre-contemplation through exploration by which organizations ultimately decide to adopt the innovation (programme/model/process). Implementation describes the formal strategies to promote the integration of innovations into existing practices. Assimilation is the informal process by which, over time, innovations become part of routine ways of doing things. Assimilation can be characterized as (a) transformation when there is high fidelity to the model and the organization adjusts its functioning to fit the assumptions of the model; (b) customization when the model is adapted to the context and the organization adjusts its practices; (c) loose coupling whereby the innovation is adopted only superficially, while the functioning of the organization remains largely unaffected; or (d) co-optation whereby the innovation becomes captured and distorted to reinforce existing organizational roles and power structures [21].

## Methods

### Study aim and design

We adopt a longitudinal multiple case study approach to understand the dynamic nature by which three coproduced innovations intended to address the needs of vulnerable populations were adopted, implemented and assimilated [22]. Case study research is well suited to studying contemporary phenomena in their real-life contexts, and theory is often adopted to focus the analysis, allowing the theory to be augmented or revised based on emerging findings [22]. To meet the criteria of being a ‘case’, an innovation had to be underpinned by a coproduction model involving structurally vulnerable populations in the design, management, delivery and/or evaluation of a public service that has advanced through these phases. Concepts from the CFIR, DOI and assimilation frameworks described above informed the case study protocol, semi-structured interview questions, data extraction and coding.

### Case selection

The three cases were selected through the networks of the investigators to illustrate how coproduction involving vulnerable populations can be advanced in different contexts: the region of Jönköping, Sweden striving for better patient outcomes and experiences by tailoring care to the needs of people with multiple complex needs (Case 1 – ESTHER); the city of Dundee, Scotland aiming to advance the recovery of people with mental illness through greater collaboration with those with lived experience and among service organizations (Case 2 – Making Recovery Real [MRR]); and a rural and an urban branch of a national community mental health organization in a Canadian province that adapted the English Recovery College model of coproduced educational programming to support the recovery of people with serious mental illness (Case 3 – Canadian Mental Health Association [CMHA] Manitoba and Winnipeg and CMHA Central branches’ Learning Centres in Manitoba, Canada) (see Tables 1, 2 and 3).

**Table 1:** Case features – Case 1 ESTHER

Location	Jönköping County, Sweden
Administration	Jönköping County Council
Structurally vulnerable group served	Initially older patients with complex care needs, then expanded to patients of any age with complex needs
Coproduction approach	Initial focus on radical customization of an Esther’s journey through health, and evolved over time to become coproduction with patients through the involvement of Esthers in storytelling cafes, meetings and projects, to provide feedback based on their experiences
Guiding question(s)/aim	• What is best for, or important to, Esther? • Who needs to cooperate to fulfil this? • What do we need to improve?
Inclusivity principles	• Focus on care coordination in complex needs population • Openness, transparency • Joy, “serious fun” • Driven by value (not money) • Reconnection to original healthcare values • Balancing power
Addressing structural vulnerability	The objective of ESTHER is the coordination of those care needs identified as most important to patients themselves. Vulnerable populations, specifically those with chronic diseases, are those expected to benefit most from ESTHER as these groups are more likely to ‘fall through the cracks’ of the system, given their complex care needs across the health and social care systems

**Table 2:** Case features – Case 2 Making Recovery Real

Location	City of Dundee, Scotland
Administration	Initially, a collaboration of 10 public, voluntary and community organizations, led by the local Third Sector Interface organization
Structurally vulnerable group served	Initially, people with lived/living experience of serious mental illness
Coproduction approach	Coproduction is at the heart of initiative from the start. The objective was to centre lived/living experience of recovery when bringing service users and carers together with health and social service providers in the community of Dundee to determine how to make recovery real in this community. Adopted an asset-based approach that discovers capacity within individuals.
Guiding question(s)/aim	What would make recovery real in Dundee?
Inclusivity principles	• Rooted in mental health recovery principles (connection, hope and optimism, identity, meaning, empowerment) • Sharing lived experience through recovery stories • Aim is transformational system change within and by communities • Moving beyond the medical model of service delivery
Addressing structural vulnerability	Collaborative conversation approach to create an environment through sharing recovery stories in which people with lived and professional experience can work together to identify what is possible. Focus on encouraging involvement from underrepresented communities.

**Table 3:** Case features – Case 3 CMHA Learning Centres

Location	Winnipeg and Central Manitoba regions, Manitoba, Canada
Administration	CMHA Manitoba and Winnipeg, and CMHA Central branches
Structurally vulnerable group served	Initially, people with lived/living experience of serious mental illness but expanded to whole community with interest in living well
Coproduction approach	Coproduction, co-development, and co-learning are at the heart of the Recovery College model. The aim is to be peer-centric and peer-led and to foster collaborative and authentic relationships with students, so they have meaningful involvement. Social or co-learning is active and involves looking at topics from both the professional and experiential lenses, interaction with others, learning from their experiences, and contributing to the learning of others.
Guiding question(s)/aim	Provide educational programming to foster recovery and living well principles
Inclusivity principles	• Rooted in mental health recovery principles • Living well for all • Inclusive educational approach • Courses are open to anyone interested in participating/learning
Addressing structural vulnerability	The coproduction approach fosters a fundamental relationship shift between staff and students. The instructional climate creates a sense of community that is absent for many structurally vulnerable individuals. Service providers develop skills in coproduction, well-being, and recovery and become more open to innovation.

The study team were familiar with each of these cases and were confident in having good access to them over time. Additionally, their different national contexts offered the opportunity to consider macro-level factors. While each of these countries’ health and social care systems are largely publicly funded, funding is the responsibility of different levels of government (municipal, provincial and/or national) and services are administered and delivered primarily by local governments and/or designated authorities (see Table 4).

**Table 4:** Health and social care system features

System features	Case 1 – ESTHER	Case 2 – Making Recovery Real	Case 3 – CMHA Learning Centres
Primary funding model	Public	Public	Public
Primary funder(s)	Regional county councils, and local municipalities	National government	Provincial government
Service delivery	Regional health authorities, regional county councils and local municipalities	NHS Scotland health boards and local authorities/councils	Provincial and regional health authorities

### Data sources and collection

Data sources include relevant academic and grey literature identified through electronic searches and/or recommended or shared by local gatekeepers and key informants to inform the background case context for the individual case analyses, and the interview guides (see Table 5, and Table S1 in Additional file 1 for more details). Research team members (GM, JG, GR, NV, PH, SC, SS) conducted 45–60 minute long semi-structured interviews in person or online between November 2019 and August 2021. To help understand the history and context of each case, key informants were strategic decision-makers and programme managers affiliated at the time with the organizations leading, participating in or supporting the local initiatives, and who were familiar with the history of how the coproduced innovations emerged, their developmental timeline and coproduction’s role in the overall system.^1^

**Table 5:** Sampling frame

Case	Coproduced innovation start year	Interviews conducted	Number of informant interviews	Number of documents reviewed
1 ESTHER	2006	11/2019 to 12/2019	2	6
2 Making Recovery Real	2015	12/2020 to 05/2021	4	8
3 CMHA Learning Centres	2017	01/2020 to 08/2021	8	18
Total			14	32

The interview guide questions probed about this history with a focus on the contextual factors that influenced adoption and implementation and the extent to which coproduction has been assimilated into routine practice. Data were gathered through investigator field notes, the audio-recording and transcription of interviews, timelines, hand-written notes and/or audio-recordings of team meetings to capture member checking with local collaborators, and case team memos of decision points.

To maintain participant anonymity, participant codes are used in the text, identified by a location code (for Case 1, JKG = Jönköping, Sweden; for Case 2, DND = Dundee, Scotland; for Case 3, OTH = Other [for example, national, international informants], PLP = Portage la Prairie, Manitoba, Canada; WPG = Winnipeg, Manitoba, Canada), and a participant number (that is, 01, 02, 03 and so on). For example, an informant from Dundee could be DND-03. Note that the perspectives of service providers and people with lived experience of structural vulnerability were not the focus here but are considered in subsequent waves of our data collection to understand their experiences of coproduction in practice.

### Data analysis

A common coding framework was developed iteratively to capture factors and processes influencing adoption, implementation and assimilation by combining elements of the theoretical frameworks to remove overlap and promote consistency of understanding when coding and interpreting the data. Table S2 presents this in more detail (see Additional file 2).

The initial data extraction was performed by the research team affiliated with each case, and the project research coordinator worked with the local research coordinator for each case to ensure consistency across cases. Documentary evidence analysis primarily informed our understanding of the historical context and overview of each case. All data were coded and analysed using a deductive approach; a common coding scheme and thematic analysis were employed, respectively, based on the theoretical propositions and concepts in the CFIR and DOI models, and allowing for emergent themes, particularly in relation to the coproduction context [22]. A visual timeline was created to understand the initiation and growth of coproduction in each case. Interview data was triangulated with documentary evidence and field notes. Analysis proceeded on a case-by-case basis, followed by a cross-case analysis.

### Qualitative validity and reliability

The research team comprised four members who were familiar with one of the three cases prior to the study (the ESTHER case), as well as eight members who were not familiar with any of the cases. One member of the team had been closely involved with the development of the ESTHER case over a long period of time. The use of a common and detailed case study protocol and data management system, central and local research coordination by case, monthly investigator meetings and tri-annual full team meetings including collaborating organization representatives were strategies used to enhance qualitative validity. The common coding framework and frequent team discussions helped to ensure consistency and enhanced reliability. Data were triangulated across sources, the analysis was triangulated across investigators and theories, and member checked at various stages with the full team of investigators and collaborators [23].

### Ethical considerations

Research ethics clearance was obtained from the relevant academic research ethics boards (McMaster University Research Ethics Board [MREB Project ID 2066], Aston University Ethics Committee [Rec Ref #1611]; King’s College London Research Ethics Office [Reference Number MOD-19/20-17350]; SingHealth Centralized Institutional Review Board [CIRB Ref# 2020/2341]; and Swedish Ethical Review Authority [Etikprövningsmyndigheten, Dnr 2019-06373]), and in light of this, ethics review was waived by the boards of the collaborating organizations (Canadian Mental Health Association, Manitoba & Winnipeg branch, the East of Scotland Research Ethics Service). Participants received letters of information outlining the study objectives, protocol and risks prior to consenting in writing. Data were collected and stored locally and shared across sites as anonymized, encrypted and password-protected files.

### Findings

**Fig. 1:**
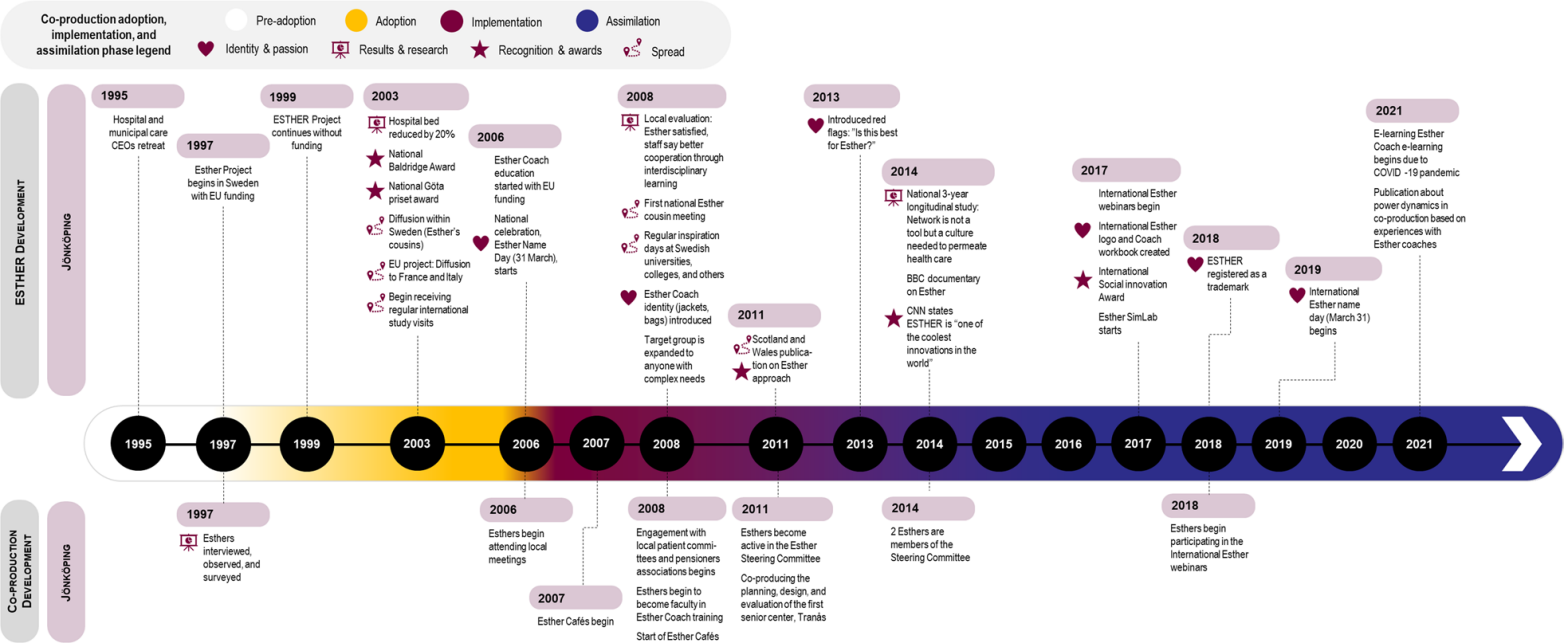
Case 1 ESTHER coproduction adoption, implementation and assimilation timeline

**Fig. 2:**
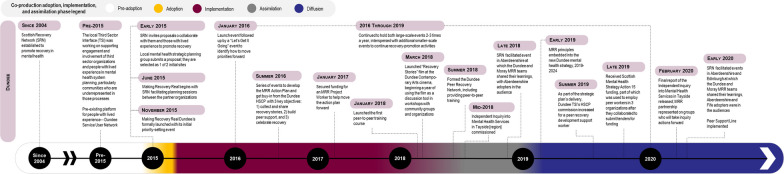
Case 2 Making Recovery Real coproduction adoption, implementation and assimilation timeline

**Fig. 3:**
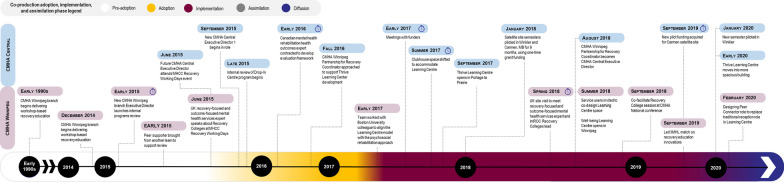
Case 3 CMHA Learning Centres coproduction adoption, implementation and assimilation timeline

We outline the historical context and analysis of contextual factors influencing adoption and implementation, discuss assimilation by case and then present a cross-case analysis. Tables 1, 2 and 3 above capture the key features of each case, Figs. 1, 2 and 3 summarize the adoption, implementation and assimilation timelines, and Tables 6, 7 and 8 summarize the cross-case analysis.

### Case 1

Historical context: ESTHER is a complex system of public health and social care services run by 13 municipal councils in Region Jönköping County, Sweden that has brought intersectoral health and social care providers together since the 1990s to increase coordination and to redefine service experiences around the needs of the person receiving the services. In a context of restricted public sector funding, ESTHER began in 1997, initially for 2 years, with the aim of finding ways to meet population health needs using approaches other than increased hospital bed capacity. Hospital leaders in Region Jönköping County aimed to transform ways of working and to prevent hospital admissions through what informants called “radical customization”, which considered the needs of individual patients using a bottom-up change process referred to as health process re-engineering. This approach 'shadowed' a patient with complex needs through their health service experience journey and included interviews and surveys with patients, staff and government officials and observations of care encounters and processes to gain new insights into what was needed to improve the system from the patient perspectives. Storytelling of the experience of 'Esther', a persona of an elderly person with complex health needs, actualized this process, pointing out what needed to be done differently by demonstrating the importance of focusing on the experience of the person receiving care. The lessons learned from ESTHER fuelled health and social service-wide change, including coproduction with patients beginning in 2006 through patient roles on advisory committees and councils, and has expanded to include initiatives such as ESTHER cafes, ESTHER coach training and ESTHER family meetings, among others.

Adoption: In the ESTHER case 'adoption' of coproduction was an emergent phenomenon that took place over a 10 year period as ongoing improvement efforts, aimed from the outset at better capturing the lived experience of people with complex needs, evolved in terms of how their perspectives were incorporated in design and decision-making. This initially began with interviews and shadowing patients and bringing staff on board with this approach, until by 2006, Esthers became more directly involved in coproducing system improvements. In the *internal context*, healthcare process re-engineering efforts since the 1990s centred on the question of “What is best for Esther?” and demonstrated the importance of person-centred care and emphasizing the experiences of the person in need of complex care, laying the foundation for a coproduction approach to emerge. In the *external context*, system-wide efforts by health and social leaders to create a system map led to ESTHER becoming more than a health quality improvement project but rather a health and social systems-wide movement. From a *process* perspective, the initial project’s evaluation results indicated a 20% reduction in hospital beds, an achievement that earned recognition in the *external context* through two national awards. As project funding ended, the benefits of the ESTHER philosophy were recognized, and ESTHER transitioned from a project to a 'network' without funding. Over the next few years, the ESTHER Network further developed as 'cousins' emerged across Sweden, and the approach was adopted in other countries, including Italy, England, Scotland and France.

By 2006, ESTHER in Sweden transitioned toward adopting coproduction approaches that actively invited participation of people with lived experience expertise (Esthers) in coproducing ongoing innovations; however, this *process* was emergent and not uniform. The flexibility of a guiding philosophy was a key *feature* that enabled this emergence of innovation in the coproduction approach. By this time, some *individual* system leaders had come to recognize that keeping the focus on value and what is best for the person being treated in their daily lives would lead to better results than a preoccupation with resources and cost cutting. ESTHER had transformed relationships *internally* in hospitals to team-based (doctor‒nurse) coleadership and *externally* across the region via interorganizational collaboration between hospitals, primary care, community care and social care to improve Esthers’ care journeys. These collaborative ways of working were preparation for collaboration with Esthers, helping to create receptivity among senior leaders to coproduction. Nonetheless, at this stage of adoption there was still some internal resistance, particularly at middle management and staff levels, as Esthers began attending and sharing stories about their experiences at leadership meetings.

“I think one of the most important decisions was to take patient in the room. In addition, there was a lot of resistance”. [JKG-01]

Implementation: Once the decision to work directly with Esthers was taken, the implementation of coproduction has continued to unfold, albeit unevenly and opportunistically. Around this time, factors in the *outer context* shaped ESTHER’s continued development, as Esthers became increasingly present in local patient committees and began to participate in and influence the ESTHER steering committee. While ongoing primary care reform was a distraction for many health service managers, an external network of Esthers developed from different programmes across municipalities, and annual ESTHER 'family' meetings were held, where Esthers could convene to share experiences and ideas, strengthening the grassroots support. ESTHER was again gaining international recognition, becoming the subject of a BBC documentary film and being declared “one of the coolest innovations in the world” by CNN.

In the *internal context*, further developments included the creation of internal structures that were funded to support greater involvement of patients with multiple vulnerabilities in coproduction activities: The ESTHER Competence Center, training healthcare teams to follow the ESTHER philosophy, and ESTHER Coach quality improvement training programmes for approximately 30 health and social service providers to become new ESTHER Coaches each year, and with growing numbers of Esthers as faculty. Key *features* of the approach were supportive of grassroots growth. Coaches developed innovations on an ongoing basis with input from Esthers, and health and social service providers remarked that the ESTHER philosophy takes them back to the reasons they entered their professions. At the same time, the bottom-up nature driving innovation continued to be threatening to some *individuals* in senior leadership positions who were more distanced from observing the benefits.

“ESTHER is very much bottom-up. So, you are very close to ESTHER … you see what’s going on and what you can do better. The steering is from the bottom, and then the managers got a bit threatened. I think there was suddenly too much; the movement was suddenly too big. So, people were reacting to that. …That still is a challenge”. [JKG-01]

Creative approaches have been used to foster growth despite this resistance. Small changes such as renaming committees have enabled participation by Esthers.

“We had our ESTHER Strategy Days. It was once a year that we had a really big gathering about what we are going to focus on. And we invited managers, we invited the coaches, we invited Esthers. So, one-third of the group [of 30] were Esthers and the other were working in health and social care. And, for me, that was a very big success, but it also became a threat. So, they took it away because they said you can’t have strategy day because you are not a manager. So, we changed the name. Now we have the ESTHER Inspiration Day”. [JKG-01]

The implementation process has been incremental and iterative to balance the grassroots pressure for innovation with the internal resistance to patients as equal partners, while ensuring real change results. As an example, in 2007, ESTHER cafes were introduced to connect Esthers and to identify the improvement possibilities most important to Esther. These cafes continue to be held four times per year and have attracted a wide audience, including clinicians and politicians. Esthers share their stories to help leaders and practitioners understand individual experiences, but the *process* also builds credibility: it requires a check-in with leaders and service providers about what they heard and whether that is consistent with what the storyteller feels is most important, and agreements are reached before the meeting ends about specific action(s) that will be taken to address what is important to Esthers.

“When we listen to a story, we ask the group, ‘What did you hear?’ And we are trying to confirm whether we are hearing different things than [what] Esthers really mean. So, the staff sometimes think, ‘This is very important’. But when we give that back to Esther, she says, ‘Well, that’s not so important for me. For me, this is important’. So, the ESTHER cafe is an activity to identify improvement possibilities. That’s one of the activities”. [JKG-02]

Assimilation: By 2016, ESTHER had evolved from being a network to becoming assimilated as a mindset – the central concept driving innovation in the system in the Jönköping Region. By this time, the decision was made to withdraw funding specific to ESTHER other than to support coach education and to have no single person responsible as leader, as it is intended to be fully assimilated as part of the normal way of working. At the same time, without dedicated funding and leadership, questions remain about sustainability.

“As I said, it is a mindset. Now it is implemented in these programs – the question: ‘What’s best for Esther?’– you will find you can’t find one person who is responsible for ESTHER in Sweden, but there is a programme group and the programme group is trying to find out ways how to spread it in the whole region, because we have some difficulties there. It’s a mindset and it should be part of the daily work. And we are getting there. I think it’s very much dependent who is leading all these kind of leadership programmes, and do they really take the ESTHER philosophy to heart?” [JKG-02]

At this point, all steering groups were removed, being seen as no longer necessary. This removal of infrastructure (formal structures, funding) initially concerned committed leaders, with a risk of co-optation of the ESTHER concept without true adherence in practice. However, there was a widespread sense among interviewees that the ESTHER philosophy has been assimilated as a core value that continues to influence all activities, permeating the culture to become the routine practice in Jönköping.

“It’s a very normal mindset in one of our hospitals to ask the question, ‘What’s best for Esther?’ That’s just a normal way of working and people are just using that word and that question”. [JKG-FL-01]

See Fig. 1 for a summary of the Case 1 adoption, implementation and assimilation timeline.

### Case 2

Historical context: Making Recovery Real gives people with lived experience of mental health difficulties the opportunity to be at the centre of decision-making, service design and practice development in the community of Dundee, Scotland by changing the terms of the dialogue about recovery, mental health and well-being. It began in 2015 as a collaboration of 10 local public, voluntary and community organizations who responded to a call from the Scottish Recovery Network (SRN) to work together to take a new approach to improve the experience and outcomes for people living with mental illness. Initially, the partner organizations endeavoured to develop and deliver more recovery-focused policies and practice by centring lived experience in answering the question: “How can we make recovery real in Dundee?” They brought together interested people, including those with lived experience, at collaborative cafes; a series of events where priorities and accompanying actions were identified, and where participants were equal contributors to the process and its outcomes. To foster the integration of lived experience into system design and practice, the priorities identified were to (i) collect and share recovery stories so that lived experience is at the core of service design, delivery and practice; (ii) develop peer support roles and training; and (iii) celebrate recovery [24].

Adoption: In the *external context*, the mental health system remained dominated by the medical model, a lack of system innovation and acute services prioritized over community services. Yet, recent Scottish health and social care system integration has supported partnership working. Simultaneously, SRN, a national voluntary organization established in 2004 to promote recovery principles within the mental health system, was shifting from working with the National Health Service towards building coalitions of change within communities and a whole-systems approach to promoting recovery. SRN solicited proposals from local groups and organizations, offering their support for community-based collaborations that would involve people with lived experience in developing local initiatives to support mental health.

Factors in Dundee’s *internal context* also converged to support a proposal put forward to SRN for an innovative approach. First, the Dundee Third Sector Interface (TSI), which supports the representation of third sector organizations in local authority planning, had been working to better involve people with lived experience in mental health system planning, and meetings with their network members were becoming more recovery focused. A recent inquiry into mental health services and a fairness commission on poverty (a longstanding local issue) also motivated the local council and Health and Social Care Partnership (HSCP) to take innovative action focused on prevention versus mitigation.

“And I think the Health and Social Care Partnership realized that they needed to do more than mitigation … they have been really, really clear on the need for new ways of doing things for about the last 10, 15 years”. [DND-02]

Furthermore, Dundee City was preparing to develop a new mental health strategic plan and, in the hope of influencing the strategic priorities and the future approach to engagement locally, the TSI brought partners from across community services, the local authority and representative groups who had been attempting to make change in the system to submit a proposal for SRN’s support. *Individual leaders* from within the partner organizations, motivated by their own lived or professional experience, were drawn by the *innovation’s features*: to support any concerned citizen to contribute their inherent resources through meaningful involvement and an asset-based approach:

“… So lived experience is essential, bringing people together, involving everybody who wants to be involved in each aspect of the process; so, firstly in agreeing what it is they want to achieve, then in making sure that it is carried out, also in having an actual role in actively carrying it out, so not just identifying things other people should do but having a vested interest and an active contribution to the activities that are going to be – whatever it is that’s going to be done differently, basically”. [DND-04]

SRN acted as a change agency, helping to alleviate tensions among the coalition and supporting their process of exploring the opportunity and submitting a successful proposal.

Implementation: First, SRN helped to bring the *individuals* involved together to establish a shared vision for the process among the local integration bodies (TSI and HSCP) and a TSI-supported service user network, reducing competition among the service provider partners. Within the *inner context* of the partnership, there was a commitment to coproduction processes and peer support as a critical opportunity to incorporate more lived experience into the mental health system. Despite these efforts, some of the original partners could not align themselves with the experience-led approach and discontinued their involvement knowing they could return at any time. Undaunted, the remaining partners proceeded by working with the “willing”, beginning with increasing local knowledge of recovery approaches and exploring what recovery meant to local citizens.

“… at the very start, it was a case of, ‘Right. We don’t really know where we want this to go. And actually, are we the ones to be dictating where this should go? No, we’re not. What’s most important is that we’re listening to people with lived experience, people on the ground, and they should be the ones that are telling us what needs to be changing’. So from the beginning, the sort of first step was looking at how we can engage with local people. And we were really keen to make sure that it was meaningful … And we thought this involvement can’t be tokenistic. People need to be on board, and it needs to be collaborative from the start”. [DND-05]

To build connection and trust between participants while shifting to a peer-led approach, the implementation *process* involved facilitating a series of coproduced, discussion-based events where people with lived experience were invited to be involved in all stages from planning and executing the events, to identifying and achieving priorities. The role of professionals shifted to “being on tap, not on top” [DND-02]. SRN provided developmental support to the Dundee partners to deliver the events, the *features* of which were welcoming and inclusive, avoiding formal presentations in favour of fun, health-promoting activities that allowed community members to feel heard, and demonstrated alignment with their own ideas and values.

“… what we did—and I would say I think that really set the tone – was rather than have lots of presentations, what we did was, at the event, we welcomed everybody, but we invited lots of the groups to run taster sessions of the things they did. So, that actually brought a lot of people with lived experience because they were coming along to demonstrate their finger painting. There was hula-hooping. There was wellness action planning. There was how to sleep well [sessions]. And in every corner of this venue, there was little groups of people who were painting pebbles, things like that. And then in the afternoon, we had a big conversation happen, world café style. And the sort of comments we got from people were, ‘I felt this was my event. This was for me. It wasn’t for them, the professionals’”. [DND-02]

From these discussions, it emerged that understanding local experiences of personal recovery was the most preferred and effective conveyor of local knowledge and motivator for change for the range of stakeholders. Storytelling became the primary vehicle for relationship building. Peoples’ stories were compiled into a film that premiered at a well-attended, prestigious 'red-carpet' event at a local cinema house, and subsequently became a tool to foster collaborative conversations at engagement events.

“And the film galvanised things and I think because we’d moved beyond that individual telling their story to having a 20 minute film of people reflecting on recovery, which is quite different from telling a story, say, of illness”. [DND-02]

The film drew strategic attention to MRR. This culminated into a consensus to embed recovery, backing for continued peer support and recovery work into the new Dundee Mental Health Strategy and accompanying action plan.

Assimilation: The MRR partner organizations have adopted a peer-led approach to their efforts to promote mental health recovery going forwards. Partners are also now far more involved in collectively determining the distribution of funding through the HSCP and in designing new mental health services.

Locally, the MRR approach has also been included in the Dundee Mental Health Strategy, granting the third sector more influence and collective power in local health and social care planning. The adoption of the MRR approach by the Dundee HSCP has strengthened the importance of mental health locally, dovetailing with the recommendations of the independent inquiry on poverty. At the national level, a Scottish government funding programme to increase the number of mental health workers in community-based services provided an opportunity for the HSCP to fund additional peer support roles, a key initiative within MRR.

Overall, the MRR partnership can be said to have had a transformative effect locally. It has led to better working relationships between providers and continues to drive progress. Furthermore, lived experience is being built into the system infrastructure through actions prioritized in experience-centred collaborative conversations: expansion of the local peer recovery network, development of peer support roles, implementation of peer-led services, peer support training provision and building recovery awareness. A key feature of ongoing progress has been that lived experience partners have been able to move in and out of active participation roles throughout the process, as their recovery journeys and contexts have allowed.

“There was that sense of collaboration that continued ... We kind of all came together to discuss how we felt our organizations could contribute to that bigger picture and the strategic objectives moving forward, and not just the strategic objectives in relation to Making Recovery Real but the wider kind of city and what they were looking for in relation to the local mental health strategy and the city plan”. [DND-05]

Participants describe the process as a difficult yet joyful and rewarding journey. For some organizations, the introduction of the MRR approach has motivated significant recovery-oriented change in their values and structure, further cementing system-level impact.

“Making Recovery Real has really been – I suppose we’ve adopted the principles and approaches … We try to adopt those as far as possible in all of our work. And we don’t badge it all Making Recovery Real, but we use the learning from it, I would say, in everything we do now, everything in the programme”. [DND-04]

See Fig. 2 for a summary of the Case 2 adoption, implementation and assimilation timeline.

### Case 3

Historical context: CMHA Learning Centres began development in Manitoba in 2015 as a coproduced adaptation and renaming of Recovery Colleges, which originated in England in 2009 with a focus on people with lived/living experience of serious mental illness. The aim of Recovery Colleges is to bring the lived experience of people with mental illness and other community members together with professional expertise to locally plan, develop and deliver educational courses about mental health and recovery, with the aim of empowering people to support their mental health and well-being. The concept of recovery education originated in the USA [25, 26], and before adopting the Recovery College model, CMHA Winnipeg had offered psychosocial rehabilitation (PSR)-based recovery education since the early 1990s. In 2015, the CMHA Winnipeg branch leader conducted an internal evaluation of this programming, which suggested that improvement was needed to meet the psychosocial health and well-being needs of the community. Around the same time, the new leader of the CMHA Central branch in Portage la Prairie, Manitoba sought a fresh approach to its clubhouse programme, a mutual support drop-in centre, in response to member feedback. Leaders and service users of both branches embraced the Recovery College and coproduction approach to better meet client needs. CMHA Learning Centres build on the Recovery College principles, with the programming and the target audience expanded to promote living well among the broader population, as well as recovery education for people with lived experience of mental illness. The CMHA Central branch’s Thrive Learning Centre and the CMHA Winnipeg and Manitoba branch’s Well-being Learning Centre opened in September 2017 and January 2018, respectively.

Adoption: In the *external context*, the national policy context was supportive of a recovery and well-being approach; it was the focus of consultations over the 2008–2012 period prior to the release of Canada’s mental health strategy [27]. This enabled Manitoba bureaucrats to pressure provincial government leaders to cosponsor a 'Recovery Days in Mental Health' conference held in Winnipeg in June 2015. An English Recovery College champion was a keynote speaker and sparked interest in the model among CMHA branches in Manitoba. The Winnipeg Regional Health Authority (RHA), the major funder of the Winnipeg CMHA branch, also supported recovery and mental health promotion approaches. Informants reported that Manitoba’s culture of innovation and solidarity, with its many small rural communities, also aligned with the coproduction philosophy of inclusive innovation.

In the *internal context*, the Recovery College model resonated with existing branch cultures of deep commitment to recovery-oriented work and strong peer support foundations. CMHA’s federated structure allowed each branch autonomy to develop its own programming, with support from a national office. Attractive *innovation features* were the existing evidence base, emphasis on lived experience through coproduction in course development and facilitation, opportunity for student skill building, and flexibility to accommodate local needs and strengths. The instructional climate was also appealing, as it could offer people with lived experience a sense of community and could promote their self-efficacy and confidence while reducing the power imbalance and fostering relationships between staff and students. The Recovery College model could also offer a more immediate response in terms of educational support to people needing care and facing long wait times for traditional services.

“I would say there’s probably many other things besides instruction. I think there’s relationship-building that happens so there are connections between students and between the facilitators and the learners. It’s the development of a space that allows for people to develop skills that are unrelated to the content. So, people also learn skills like sharing in a group context, so confidence-building, self-efficacy. When you can cultivate a skill in one area, you build confidence, and you start to believe that you have the ability to learn and to develop new skills. So that sense of self-efficacy is very integral to the recovery and well-being journey”. [WPG-02]

The importance of *individual characteristics* was demonstrated as passionate leaders in the Winnipeg and Central branches who were committed to advancing upstream mental health promotion and PSR were impressed by the model and together, they researched it further to inform adoption decisions. The coproduction *process* aligned with CMHA’s “nothing about us without us” approach and could foster a sense of ownership. In both branches, the name Recovery College was changed to Learning Centre during the adoption process, which better resonated with community and agency participants.

Implementation: In the *external context*, in early 2017, CMHA Winnipeg and Central branches met with CMHA National to implement Learning Centres. Although no new funding was made available by the RHAs, philosophical support enabled the repurposing of existing funding for recovery education and peer support. In 2018, CMHA National and CMHA Winnipeg leadership visited England to meet recovery-focused mental health services experts and to see the model in action. This visit was crucial in fostering strong relationships between the model initiators and CMHA leaders who discovered common visions to widen the target audience to anyone in the community interested in mental health issues, thereby making mental health a universal concern and promoting a living well approach. Collaboration with an Ontario-based psychiatric hospital, with similar values and interest in Recovery Colleges, supported programme evaluation to produce evidence of effectiveness.

*Internally*, the Winnipeg and Central branches collaborated on initial model and course development, and took a staged approach to opening their Learning Centres. In the Central branch, where resources were tighter and there was a large geographic area to serve, creative approaches to leverage local support and assets were used. Health professional placement students supported the small branch to prepare for launch and in doing so, encouraged staff buy-in. Another peer service organization provided funding support and this, along with community grants, covered staffing, technology, social marketing and other costs that are traditionally not eligible for provincial funding.

“[A] critical moment would be the establishment of a partnership. I think that was a critical moment. I walked away and I know my staff did, too, with an immense sense of relief after I could tell them that [a peer Manitoban mental health community organization] was on board to help make this a reality”. [PLP-22]

The Winnipeg branch also leveraged internal resources, including an existing peer support group whose members assisted in developing the first five courses.

“And so we actually relied on some communities that existed within our CMHA. So we had a group of individuals who are peer supporters to one another. They had taken our workshops in the past. And then they created, on their own, their own support group, and designed that support group based on their needs and on an educational focus. So we actually asked them if they would be our initial coproduction group”. [WPG-04]

The passion of *individual* CMHA staff and leaders, many with their own lived experience, made them champions who demonstrated their commitment to valuing expertise derived from lived experience. These individuals also helped build the external linkages with organizations and key people both nationally and internationally. *Innovation features* allowed for initial small-scale implementation, leveraging local assets and community strengths before expanding further. The flexibility to offer “something for everyone” and promote “living well in your community” garnered broad interest and unanimous buy-in from community members. The flexibility of the model also allowed the Winnipeg branch to retain PSR influences from their colleagues at Boston College.

The collaborative coproduction *process* fostered a sense of ownership, friendship building, balance across perspectives and acceptance within the classroom. This affirming process allowed room for creative input and for trial and error, with the process itself evolving to become more effective over time. It also facilitated the expansion of course offerings, as students were encouraged to lead future course development. Accompanying changes to the physical space and staff roles helped in welcoming the whole community, meeting the needs of vulnerable groups in society and addressing access barriers.

Assimilation: The Central branch has been unable to coproduce new Learning Centre material during the COVID-19 pandemic, yet it continues to offer its existing content. The Winnipeg Learning Centre was able to shift to virtual and then hybrid online and in-person coproduction activities, while ensuring fidelity to the core Recovery College principles.

“And some of the other things that are in the fidelity assessment are: Are you recovery-focused? Are you community-focused? Are you collaborating with the people who are consuming your services? So, it’s a really easy fidelity to conform to but also have room to be kind of creative because they’re not dictating what courses you should have. The fidelity is that you provide courses”. [WPG-04]

In Winnipeg, the Learning Centre continues to expand and evolve, and is reported to have had a gradual but transformative impact on organizational context and values within the branch, by providing a universally accessible platform that demonstrates the value of engaging people with lived experience at every step. The coproduction approach to course development has ensured that content remains current and relevant through creativity, diversity and responsiveness to people’s needs. Leaders’ commitment to the model and ongoing evaluation to ensure it is meeting local needs have supported wider assimilation of coproduction approaches in other branch programming as well. New leadership in the Central branch has expressed the desire to revive the Learning Centre’s coproduction activities. 

See Fig. 3 for a summary of the Case 3 adoption, implementation and assimilation timeline.

### Cross-case comparison

Adoption: Shifting ideas in the public policy realm and supportive external change agents created a conducive *external context*. In Cases 1 and 2, shifting ideas pertained to interprofessional and intersectoral collaboration and in Case 3, national and provincial discussions about a recovery and well-being orientation were important precursors to coproduction with people with lived experience. *Internally*, tension for change was evident in all cases; however, the process by which this unfolded differed, as a natural progression of ongoing improvement efforts in Cases 1 and 3 and as a deliberate response to an opportunity created by an external change agent for local system-wide transformative change in Case 2. In all cases, passionate *individuals*, many with their own lived experience, and a philosophical approach that resonated deeply and widely was a core *feature* leading to adoption (see Table 6).

**Table 6:** Cross-case comparison: adoption

CFIR/DOI element	Case 1 - ESTHER	Case 2 - Making Recovery Real	Case 3 - CMHA Learning Centres
External context	• Early ESTHER work normalized collaboration between health and social leaders • National awards created enthusiasm to continue to build on work to date	• Health and social care integration legislation fostered collaboration • External change agent shifting towards local coalitions for change created opportunity for mentorship	• Changing policy ideas at the national and regional levels provided philosophical support • Conference promoted recovery, introduced English Recovery College knowledge purveyor • Facilitated by change agent organization, and local and international networks
Internal context	• ESTHER had shifted medical relationships to coleadership • Internal management resistance to patients in decision-making remained	• Tension for change within local mental health service provider collective • Opportunity to influence the new mental health strategy	• Model values well aligned with organizational culture • Evaluation showed gaps • Culture of innovation and solidarity in the region
Individual characteristics	• System leaders realized a focus on value and what is best for the patient leads to the best outcomes	• SRN leader with a vision for change and community development approaches	• Visionary branch leaders also regional leaders in recovery, mental health promotion and community development • Existing peer staff to support
Innovation features	• Flexible philosophy enabled participation of patients (Esthers) in coproducing ongoing innovations	• Opportunity for local network to receive support to centre lived experience in the identification of local needs and solutions	• Recovery College model evolved existing philosophy, aligned with CMHA values and was adaptable to local context
Process	• Initial evaluation showed a 20% reduction in hospital beds • Incremental approach to introducing Esthers to venues of greater strategic importance	• TSI facilitated proposal development and building trust between partner organizations	• Coproduction processes aligned with aim to be community led • Agreement between two branches and national office to move forward in Manitoba

Implementation: In all three cases, building local partnerships and/or networks in the *external context* was integral to implementation. These partnerships and networks helped to overcome *internal* resistance within existing power structures (Case 1), created a community coalition that could move forwards in the face of resistance within traditional mental health services (Case 2), and offered material support and expertise to support implementation (Case 3). In Cases 1 and 2, there was no 'programme' per se, rather a philosophy steered by guiding questions, and in Case 3, the Recovery College model itself was designed to realize its embedded philosophy through coproduced educational programming. These *features* drove a micro-level movement for change (all cases) that was locally adapted, for example, to become “something for everyone” (in Case 3). Philosophical alignment also helped in building trust across collaborating organizations to support implementation and as a shared foundation for overcoming differences during implementation. Implementation proceeded incrementally at the grassroots level in all cases and by working with the willing (see Table 7).

**Table 7:** Cross-case comparison: implementation

CFIR/DOI element	Case 1 - ESTHER	Case 2 - Making Recovery Real	Case 3 - CMHA Learning Centres
External context	• Development of ESTHER network and national recognition helped maintain momentum despite other system priorities	• Received local political support, including representation at events • External change agent supported implementation	• Strong community partnerships with other local agencies to promote and codeliver courses • Peer organization supported with funding • External change agents supported conceptualization, implementation and evaluation
Internal context	• Structures to support Esther input developed	• Led by TSI which held a strong commitment to coproduction approach	• Philosophical alignment with organizational values and culture facilitated implementation
Individual characteristics	• ESTHER leaders committed to the philosophy • Bottom-up nature and growth was threatening to senior leadership	• Support available to build local capacity and confidence • Leads motivated by personal or professional experiences	• Passionate staff with lived experiences supported establishing and managing Learning Centres
Innovation features	• Philosophy aligned with reason people entered healthcare • Coaches became grassroots change facilitators • Incorporated “serious fun”	• Coproduction via recovery-focused events key to meaningful involvement	• Adaptability to become “something for everyone” • Lived experiences is valued as expertise
Process	• Adopted incremental, quality improvement approach to integrating coproduction • ESTHER cafes attract a broad range of listeners to hear lived experiences and identify improvements	• Worked with the willing, starting with the local integration bodies • Recovery story film became an important tool for documenting and spreading the message	• Shared vision and philosophy to overcome challenges, evolved from consultation with people with lived experience that transformed previous approaches

Assimilation: There have been different forms of assimilation across all three cases, with transformative impacts not only on the organizations involved but with impacts extending to the broader organizational and political context. A widely embraced mindset in the region, new structures and a growing international network (Case 1); impact on the local mental health strategy and continuing transformative effects on partnerships among community agencies (Case 2); and assimilation to other programmes and branches (Case 3) are some of the ongoing transformative impacts.

In Case 3, assimilation was characterized by customization, as both branches have changed the name and broadened the reach of Recovery Colleges, while maintaining fidelity to core principles. At the same time, challenges to sustaining such transformative change going forward were a concern without targeted leadership and funding (see Table 8).

**Table 8:** Cross-case comparison: assimilation

Assimilation scenarios	Case 1 - ESTHER	Case 2 - Making Recovery Real	Case 3 - CMHA Learning Centres
Co-optation	• Co-optation resisted at a number of points (for example, not participating in national project that required a name change, changing name of Strategy Days to Innovation Days rather than not hold them)	• Resisted co-optation of the approach by the health care sector by intentionally avoiding the term 'coproduction', and by not agreeing to proceed in conditions that didn’t align with the philosophy	• Manitoba Justice ministry has co-opted the anger management course, making it mandatory for some criminal offenders, which is contrary to the Recovery College philosophy
Loose coupling	• Without dedicated funding and leadership, questions about sustainability remain	• NA	• Due to lack of resources, CMHA Central Manitoba branch has been unable to continue coproduction processes during the COVID-19 pandemic, yet still offering pre-existing courses
Customization	• NA	• NA	• Name change and expansion of target group, no longer tailored to mental health recovery but to living well for everyone
Transformation	• ESTHER mindset considered fully assimilated as a mindset in Jönköping Region	• Building infrastructure for transformation through (i) establishment of a local partnership approach and commitments to priority areas and (ii) continuing to build peer-led services into the system (codesigned and codelivered) • Locally, has influenced the mental health strategy; however, political support and/or government direction unknown with upcoming leadership change	• Codesign, codelivery and colearning seen as “transformational” by students and staff • Introduction and expansion of coproduction approaches incorporating lived experiences into broader programming design

## Discussion

The analysis of these cases of adoption, implementation and assimilation of innovation demonstrates a range of factors from existing frameworks that shaped the stories of these coproduced innovations. The analysis also suggests additional considerations beyond established frameworks when aiming to engage structurally vulnerable people in coproduction activities that can help to overcome structural barriers and address power differentials in legacy systems.

Existing frameworks and models were very helpful in pointing to the interplay between the many factors operating at different levels in each context. These comprehensive frameworks provided a wide lens that was useful for thoroughly investigating different contextual elements. However, at times, this comprehensiveness made it difficult to tease out the essential causal story from our data to understand how each set of coproduced innovations emerged [28]. In our analysis, existing frameworks were most helpful when comparing across cases to identify overarching patterns, such as the influence of shifting policy ideas and external change agents in the external context during adoption and the role of community partners and network building in the implementation phase.

At the same time, particularly compelling considerations involving structurally vulnerable groups identified here were less evident in existing frameworks. Notably, there were two important differences in the** nature of the ‘programme’** in this context. First, existing frameworks suggest a predefined 'programme' to adopt; however, there was no predefined programme per se in two of our cases. Instead, change was more ideological/philosophical in nature, captured simply by a set of guiding questions (two cases) or embedded as a central feature of an existing program with lots of room for customization (one case). The central philosophy in these cases corresponded to efforts to raise the profile of traditionally marginalized voices by shifting normative paradigms about what types of knowledge (for example, lived experience) and whose voices (for example, structurally vulnerable service users) should be heard in traditional systems. Second, the process (coproduction) could not be disentangled from this essential philosophy and, in some cases, it was met with considerable resistance. Including vulnerable people as genuine partners in coproducing innovations was perceived as a 'threat' to some managers (Case 1) or to the prevailing orthodoxy of 'Quality Improvement' (Case 2).

These 'programme' features suggest a second consideration in terms of **implementation processes**. The clear intention to shift the existing power balance in systems and within organizations needed a set of resources that went beyond the capacity of any one organization. While high-level leaders with their own lived experience were instrumental in providing vision and support, the implementation process relied heavily on relationship building across partner organizations and networking at the grassroots levels rather than on top-down directives. Meaningful service user involvement was considered critical in making transformative service and system culture change, often disrupting traditional structures, networks and communication. Shared values, the development of a group-based belief system, core activities and a different relational environment and leadership [29, 30] are central to social movement theories. Furthermore, the definitive objective of stepping outside organizations within the formal healthcare system to instead derive a new way of working across many community organizations led by people with lived experiences is not clearly captured in existing frameworks, which typically speak to innovation within existing structures of power in organizations and systems.

Finally, the cases analysed here suggest important differences in **temporal dynamics** at play that were not elaborated in existing models. Consistent with concepts of change in complex adaptive systems and theories of policy path dependence and agenda setting, adoption could occur through a slow internal tension for change that built over time and culminated in coproduction as a natural evolution of ongoing improvement efforts or through seemingly sudden 'transformative' reform where a confluence of interested groups came together in the face of an opportunity to do something differently. Ideas about change in complex adaptive systems such as emergence, self-organization, adaptation, change over time, distributed control and tipping points [31], and from policy literature such as path dependence [32], multiple streams theory [33] and distributed control could be informative in this respect [34]. Our participants suggested that because each case relates to a set of concepts and principles that were collectively generated over time, there was a need to better understand this process as it unfolded.

While existing models were helpful in considering a wide range of factors to consider and recent updates suggest a movement away from concepts such as 'programme' to 'innovation' [19], the temporal, relational and power dimensions discussed here were validated by our collaborators as equally important considerations. Exploring these dimensions will be the focus of future work.

### Limitations and future work

This work is subject to several limitations. First, it is based on a case study of three examples of coproduction of health and social care innovations in different national contexts in the northern hemisphere. The findings may not be transferable elsewhere. Furthermore, when considering our findings in relation to the CFIR, DOI and assimilation frameworks, it is important to note that these frameworks were not specifically developed for an innovation process involving service users at all stages of innovation adoption, implementation and assimilation. However, the limitations in adopting and applying these frameworks here have led to a careful examination of what is unique to coproduction processes involving vulnerable populations. A forthcoming contribution will try to capture these unique elements and position them within the innovation, power, and social movement literatures. Finally, the analysis here is primarily based on our 'wave 1' home site findings from this longitudinal case study, and new insights may be gained from a deeper evaluation of our wave 2 and wave 3 findings. The latter pertain to processes of ongoing coproduction in practice and diffusion to other contexts, respectively, and will be analysed in forthcoming work.

## Conclusions

While our case study was extremely helpful in identifying core considerations for factors influencing the adoption, implementation and assimilation of three cases of coproduced health and social care innovations, several nuanced considerations when applying existing theoretical frameworks in the coproduction context emerged: the nature of the 'intervention' being a philosophy rather than a concrete set of steps, the intertwining of intervention and process and the need to study evolution of the intervention itself as it emerges over time, greater attention to partnered processes as disruptors to existing power structures and an emphasis on driving transformational change in organizational cultures. Future work will explore these considerations further.

### Supplementary Information

**Additional file 1. **Provides additional details about the sampling frame (that is, the organizations the interviewees are associated with, the document titles and types).

**Additional file 2. **Demonstrates how concepts from the CFIR, DOI, and compatibility gaps frameworks were incorporated into the coding framework.

## Data Availability

The datasets generated and/or analysed during the current study are not publicly available due to the study’s small sample size and the key informants’ roles as leaders within small organizations, making it difficult to deidentify their data. However, the datasets are available from the corresponding author upon reasonable request.

## Abbreviations

CFIR: Consolidated Framework for Implementation Research
CIRB: (SingHealth) Centralized Institutional Review Board
CMHA: Canadian Mental Health Association
DOI: Diffusion of innovation
DND: Dundee
HSCP: Health and Social Care Partnership
JKG: Jönköping
MREB: McMaster University Research Ethics Board
MRR: Making Recovery Real
PLP: Portage la Prairie
PSR: Psychosocial rehabilitation
RHA: Regional health authority
SRN: Scottish Recovery Network
TSI: Third sector interface
US: United States

## Footnotes

1 In some cases, these individuals also had lived experience of vulnerability that also motivated their work, but this was not a specific requirement for study participation.
