# Supplementary material for: Adopting, implementing and assimilating coproduced health and social care innovations involving structurally vulnerable populations: findings from a longitudinal, multiple case study design in Canada, Scotland and Sweden
Source: Health Res Policy Syst. 2024 Apr 2;22:42. doi: 10.1186/s12961-024-01130-w (PMC10988938; doi:10.1186/s12961-024-01130-w)
Supplement: Supplementary file 1 — Additional file 1. Provides additional details about the sampling frame (that is, the organizations the interviewees are associated with, the document titles and types). [file 12961_2024_1130_MOESM1_ESM.docx]

**Additional file 1**

| **Table S1**  ***Additional sampling frame features*** | | | |
| --- | --- | --- | --- |
| **Data source** | | Key informant interviews | |
| **Case** | **#** | **Participant code** | **Participant affiliation/setting** |
| 1 | 2 | JKG-01 | Regional government |
|  |  | JKG-02 | Regional government |
| 2 | 4 | DND-02 | Local third-sector organization |
|  |  | DND-04 | Local third-sector organization |
|  |  | DND-05 | Local third-sector organization |
|  |  | DND-07 | Local third-sector organization |
| 3 | 8 | WPG-01 | Regional service provider organization |
|  |  | WPG-02 | Regional service provider organization |
|  |  | WPG-04 | Regional service provider organization |
|  |  | PLP-03 | Regional service provider organization |
|  |  | PLP-20 | Regional service provider organization |
|  |  | PLP-22 | Regional service provider organization |
|  |  | OTH-05 | National service provider membership organization, Canada |
|  |  | OTH-08 | Independent consultancy, training and research company organization, England |
| *Total* | *14* |  |  |

| **Data source** | | Literature reviewed | | |
| --- | --- | --- | --- | --- |
| **Case** | **#** | **Author, Year** | **Title** | **Type** |
| 1 | 5 | Vackerberg, 2016 | What is best for Esther? Building improvement coaching capacity with and for users in health and social care – a case study | Research article |
|  |  | Damji et al., 2019 | What is best for Esther? What Canada can learn from the Swedish health care service | Trade magazine article |
|  |  | Gardner, 2020 | A Swedish approach to integration: Connecting the dots with a Swedish approach to integration | Trade article |
|  |  | Cribb, 2017 | The challenge of integration, in Healthcare in transition: Understanding key ideas and tensions in contemporary health policy | Book chapter |
|  |  | Kenney, 2008 | The learning journey of Jönköping County, Sweden, in The best practice: How the new quality improvement movement is transforming medicine | Book chapter |
| 2 | 9 | Making Recovery Real in Dundee, 2015 | Making Recovery Real Dundee Launch Event Report | Report |
|  |  | Making Recovery Real in Dundee, 2017 | Making Recovery Real in Dundee: Peer Recovery Opportunities in Mental Health - A co-design event | Report |
|  |  | Making Recovery Real in Dundee, 2018a | Making Recovery Real in Dundee: Using lived experience to inform the development of the Mental Health Officer Service - Report | Report |
|  |  | Making Recovery Real in Dundee, 2018b | Making Recovery Real in Dundee: A review with the Scottish Recovery Network | Report/review |
|  |  | Making Recovery Real in Dundee, 2019 | Making Recovery Real in Dundee | Handout |
|  |  | Scottish Recovery Network, 2018 | Making Recovery Real in Dundee | Video |
|  |  | Scottish Recovery Network, 2019 | Making Recovery Real in Dundee | Video |
|  |  | Sharp, n.d. | Making Recover Real: What can we do with our stories? | Report |
|  |  | Sharp, 2018 | Making Recovery Real in Dundee: A review with the Scottish Recovery Network | White paper, review/ evaluation |
| 3 | 18 | Anfossi, 2017 | The current state of Recovery Colleges in the UK: Final report | White paper |
|  |  | Canadian Mental Health Association, 2019 | Recovery College toolkit: A practical guide to support the development of Recovery Colleges in Canada | White paper |
|  |  | Crowther, et al., 2019 | The impact of Recovery Colleges on mental health staff, services and society | Research article |
|  |  | Farkas et al., 2005 | Implementing recovery oriented evidence based programs: Identifying the critical dimensions | Research article |
|  |  | Meddings et al., 2014 | Student perspectives: Recovery college experience | Research article |
|  |  | Meddings et al., 2015 | Recovery colleges: Quality and outcomes | Research article |
|  |  | Newman-Taylor et al., 2016 | The Recovery College: A unique service approach and qualitative evaluation | Research article |
|  |  | Perkins & Repper, 2017 | When is a “recovery college” not a “recovery college”? | Editorial |
|  |  | Perkins et al., 2012 | Implementing recovery through organizational change briefing 1: Recovery Colleges | White paper |
|  |  | Perkins et al., 2017 | Impacts of attending recovery colleges on NHS staff | Research article |
|  |  | Perkins et al., 2018 | Recovery Colleges 10 years on | White paper |
|  |  | Shepherd et al., 2008 | Sainsbury Centre for Mental Health policy: Making recovery a reality | Policy |
|  |  | Shepherd et al., 2010 | Sainsbury Centre for Mental Health policy: Implementing recovery – A methodology for organizational change | Policy |
|  |  | Sommer et al., 2018 | Walking side-by-side: Recovery Colleges revolutionizing mental health care | Research article |
|  |  | Toney et al., 2018 | Mechanisms of action and outcomes for students in Recovery Colleges | Research article |
|  |  | Toney et al., 2018 | Development and evaluation of a Recovery College fidelity measure | Research article |
|  |  | Australian Healthcare Associates, 2018 | Western Australia Mental Health Commission Literature review to inform the development of Recovery Colleges in Western Australia | White paper |
|  |  | Whitely et al., 2019 | Recovery colleges as a mental health innovation | Perspective article |
| Total | 32 |  |  |  |
