# Supplementary material for: Adopting, implementing and assimilating coproduced health and social care innovations involving structurally vulnerable populations: findings from a longitudinal, multiple case study design in Canada, Scotland and Sweden
Source: Health Res Policy Syst. 2024 Apr 2;22:42. doi: 10.1186/s12961-024-01130-w (PMC10988938; doi:10.1186/s12961-024-01130-w)
Supplement: Supplementary file 2 — Additional file 2. Demonstrates how concepts from the CFIR, DOI, and compatibility gaps frameworks were incorporated into the coding framework. [file 12961_2024_1130_MOESM2_ESM.docx]

**Additional file 2**

| **Table S2. Coding framework and how it includes the CFIR and DOI domains/constructs** | | | |
| --- | --- | --- | --- |
| **Node** | **Description** | **CFIR domain** | **DOI domain** |
| 1. Innovation description | A brief, generic and high-level description of what the innovation is and what it does and achieves. | | |
| - 1. Definition of coproduction | The individual or innovation’s definition of what coproduction means (i.e., their philosophy of coproduction). | -- | -- |
| - 1. Innovation’s responsiveness to  vulnerability | How the innovation serves or aims to serve vulnerable populations. | -- | -- |
| 1. Why did the innovation happen here? | Anything that is specific to how or why did the innovation come to be in the home site or adopted in the receiving site, with a particular focus on the suitability of the particular site/ location. | - Inner setting - Outer setting | - System antecedents for innovation - System readiness for innovation - Outer context |
| 1. Adoption | The factors and processes that influenced the decision to adopt the innovation (facilitators and barriers). | | |
| - 1. Innovation features | The features/characteristics of the innovation itself that influence its adoption. | -- | The innovation |
| - 1. Internal context | The internal features/characteristics within the organization that influence the innovation’s adoption. | -- | - System antecedents for innovation - System readiness for innovation |
| - 1. External context | The external influences on the innovation’s adoption. | -- | Outer context |
| - 1. Characteristics of the individuals involved in the adoption decision | The features of the individuals involved in the adoption process, that influence the adoption of the innovation. | -- | Adopter |
| - 1. Adoption process | The process of adoption, comprising a series of formal/informal decisions by organisations/individuals. | -- | - Adoption process - Linkage, in the Implementation and Assimilation phases - Communication and Influence |
| 1. Implementation | The factors and processes that influenced the formal strategies to promote the integration of the innovation into existing practices once the decision to adopt has been made (facilitators and barriers). | | |
| - 1. Innovation features | The features/characteristics of the innovation itself that influence its implementation. | Intervention characteristics | -- |
| - 1. Internal context | The features of internal organizational structural, political, and cultural contexts through which the implementation process will proceed. | Inner setting | -- |
| - 1. External context | The economic, political, and social context within which an organization resides that influences the implementation process. | Outer setting | -- |
| - 1. Characteristics of the individuals involved in the implementation of the innovation | The features of the individuals involved in the implementation process, that influence the implementation of the innovation. | Characteristics of individuals | -- |
| - 1. Implementation process | The formal strategies to promote the integration of the innovation into existing practices. | Process | Implementation process |
| 1. Assimilation | The factors and processes that influenced the informal process by which, over time, the new ideas of the innovation become part of routine ways of doing things (facilitators and barriers). | | |
| - 1. What stays the same | What parts of the innovation and/or organization remain the same as part of the assimilation of the innovation. | -- | Assimilation |
| - 1. Adaptation, reinvention, or development | What parts of the innovation and/or organization are adapted or reinvented to fit the local context as part of the assimilation of the innovation into the routine ways of doing things over time. | -- | Assimilation |
| - 1. Assimilation context | The context under which the assimilation proceeds. | -- | -- |
| - 1. Assimilation process | The process of assimilation, such as: how the assimilation process proceeded, what the key decisions were, who was involved, and how coproduction processes shaped assimilation. | -- | Assimilation |
| - 1. Effects of COVID-19 on coproduction or innovation | Responses to the interview question: How have coproduction activities been adapted to challenges, such as COVID-19? | -- | -- |
| 1. Critical moments | The really important moments as identified by the literature or participants (highs, lows, important moments) as well as: what worked well, what did not work well, and what could have been better or different. | -- | -- |
